# Use of Virtual Reality Therapy (VRT) for Home-Based Pain Management, an Observational Cohort Study

**DOI:** 10.1089/jmxr.2023.0006

**Published:** 2024-05-02

**Authors:** Megan Rumzie, Carla Figura

**Affiliations:** VA Sierra Nevada Health Care System, Reno, Nevada USA.

**Keywords:** virtual reality (VR), virtual reality therapy (VRT), extended reality (XR), pain management, chronic pain, nonpharmacologic pain management, outpatient care

## Abstract

Chronic pain is prevalent in the Veteran population at a disproportionate rate. Given the concerns for traditional pharmacologic management of pain, many are turning to nonpharmacologic alternatives for the treatment of pain. Virtual reality (VR) is an evidence-based tool, which has been demonstrated to reduce pain in hospitalized patients, and has been used for this purpose within the VA Sierra Nevada Healthcare System (VASNHCS) since 2019. Given the ongoing demonstrated benefit in this setting, these authors set to assess benefit for pain when used in an outpatient setting (demonstrations in the clinic, treatments in the patient’s home) and demonstrate safety when used outside of a supervised setting. In this analysis, pain intensity decreased by an average of 22% when comparing pre- and postimmersion pain scores, and by 12.7% when comparing baseline pain scores with the end of the analysis. Patients also reported that the use of VR reduced their stress, decreased pain, and improved their mood, and some participants were able to reduce use of their as-needed pain medications with the use of VR. These findings are limited by a small sample size; however, this study provides encouraging evidence of benefit and a framework for future, larger scale analyses.

## Introduction/Background

According to Nahin et al.,^1^ approximately 126 million adults in the United States suffer from chronic pain, and within this group up to 40 million patients face severe levels of pain on a frequent basis. This is a huge concern as it is estimated that more Americans suffer from chronic pain than those suffering from cancer, diabetes, and cardiac disease combined.^2^

Furthermore, the cost associated with treating chronic pain in the United States is staggering and approximated to be as high as $635 billion annually.^3^ In addition to the staggering financial impact, there is an equally devastating human impact associated with chronic pain, including the ramifications on a patient’s ability to work, functional status, and sense of well-being. The most frequent types of chronic pain diagnoses are musculoskeletal pain located in the back or neck and osteoarthritis.^4^ For patients younger than 45 years, chronic pain is the leading cause of disability in the United States.^5^

Chronic pain affects Veterans at a higher rate than civilians, potentially due to their service and combat exposure. Although Veterans only represent 10% of the US population, Veterans account for an unequal burden of those suffering from chronic pain with estimates of the prevalence ranging from 44% to 60% within the Veteran population compared with approximately 30% within the civilian population.^6^ Comparing male with female Veterans, it is estimated that upward of 75% of female Veterans suffer from chronic pain.^7^ Furthermore, pain is one of the most common complaints from soldiers serving in Operation Enduring Freedom (OEF) and Operation Iraqi Freedom (OIF).^8^ Unfortunately, the traditional approach to treat chronic pain (one based on the use of pharmaceuticals, including opioids) does not address the complexity of pain symptom burden or provide Veterans with a substantial level of relief from suffering due to chronic pain.^9^

Recently, complementary and integrative health (CIH) approaches have been considered an adjunct intervention for those suffering from chronic pain. Modalities such as yoga, Tai Chi, guided imagery, and biofeedback are accepted practices with the Veterans Health Administration (VHA).^4^ This feasibility study was convened to determine whether virtual reality therapy (VRT) used in the outpatient environment could help to improve pain within a Veteran population.

This modality utilizes VR head-mounted display (HMD) to create a three-dimensional experience, which may be either an actual or simulated experience. The Veteran puts the goggles on over the head and eyes and is virtually transported into an immersive space. This “immersion” combined with the “presence” of the Veteran’s attention creates the possibility for a shift in awareness, which can produce a decrease in pain, stress, and anxiety.^10–11^ VRT also provides additional benefits that include the following: it is not invasive, has little to no side effects with use, can be used anywhere, is relatively inexpensive, and can be designed to be a patient-centered experience.

Before this analysis, VRT had been used in several units in the acute care facility for the treatment of pain, demonstrating positive benefit for the Veterans who used it. Often, this led to the question, “how can I use this at home?” This prompted these authors to assess utility outside of a monitored care setting, to assess safety and utility, for exploration of future use cases, largely due to concerns for safety and efficacy, and some concerns for release of technology for use in the home without close monitoring. This treatment option was also prompted by the COVID-19 pandemic, which encouraged clinicians to provide care in unique and innovative ways to meet their patients’ needs, without unnecessary physical attendance in the health care facility, where they may have been at increased risk for infection.

## Methods

In this observational cohort study, the authors collaborated with clinicians providing pain care to identify 11 patients with chronic pain who were interested in a nonpharmacologic adjunct therapy to alleviate pain. These clinicians provided care in the physical medicine and rehabilitation (PM&R) and/or pain clinics. Professions included but not limited to physicians, nurses, pharmacists, and psychologists. Ten of the identified patients enrolled in the study. For background characteristics of the enrolled patients, see Supplementary Table S1. The eleventh patient did not enroll as a participant as they did not have internet access and thus did not meet the inclusion criteria (see Supplementary Table S2). Of the 10 enrolled participants, 6 completed the study.

The primary objective of this study was to assess the extent to which the use of VRT in the outpatient setting is efficacious for decreasing pain intensity. Secondary endpoints assessed included the safety of the use of VRT in the outpatient setting, benefit received from the use of VRT as an adjunct to present pain treatment, and the rate of return of the VRT equipment. After screening for inclusion/exclusion, all participants attended an in-clinic demonstration to ensure tolerability and signed informed consent to participate (see Supplementary Article S1), which included potential risks, benefits, protocol, and contact information. As this analysis was largely focused on desirability, feasibility, and utility, as opposed to comparison with another treatment modality, there was no blinding and all participants received the same education and instructions. Also for these reasons, participants were encouraged but not required to document endpoints as requested, in an attempt to mimic real-world outcomes as much as possible.

The study duration was 7 weeks: week 1 collecting baseline pain data without the use of VR followed by 6 weeks of VRT. During this time, enrolled patients received weekly phone calls from the VR clinical lead. During these calls, the VR clinical lead assessed for safety concerns, side effects, reported benefit, and any barriers to use. This information was obtained through both standardized questions and open-ended questions to ensure participant understanding of inquiry and reporting. Open-ended questions allowed for patients to report their experience in their own words, as well as inform clinicians of potential outcomes, benefits, or adverse effects they may not have initially intended when scripting the standardized questions. The participants were provided a pain diary (see Supplementary Article S2) to complete throughout the study, which captured pain scores, mood scores, and VRT modules completed (with pain scores before and after the immersions). Pain was rated using the Defense and Veterans Pain Rating Scale (DVPRS), an 11-point scale (with 0 being no pain and 10 being worst pain imaginable), while mood was rated using the Fordyce Emotions Questionnaire, an 11-point scale (with 10 being extremely happy and 0 being extremely unhappy). DVPRS was selected due to being the standard pain-intensity rating used within the system to ensure Veteran familiarity. The Fordyce Questionnaire was selected due to being a validated, short test to assess mood, as well as was intuitive given the participants’ familiarity with rating scales. Subjective benefits and quality-of-life improvements were elicited during open-ended questioning as well as in the Likert scale survey at the conclusion of the study. These data were cross-referenced using a dashboard feature with the reported pain scores entered within the VR device itself before and after each immersion session.

Participants received a PICO Neo2 all-in-one head-mounted VR device with preloaded XRHealth software content in kiosk mode at no cost to the patient. The Pico device is a stand-alone wireless VR HMD and, combined with preloaded content and any peripherals, makes up the VR system. XRHealth was chosen because, at the time of the analysis, it was the only VR system these clinicians were aware of that included a web-based clinician dashboard, which allowed for clinician review of device use information and validation of the reports and comments in the participant pain diaries. This system also met patient privacy requirements and expectations; information and use data were blinded to the manufacturer and were only available to the clinician. The XRHealth system included their proprietary software, which included two FDA-registered modules, Relax8 and Luna.

While the use of the FDA-registered modules was encouraged, participants were able to use any programming available within the device. This was due, in part, to the inability to restrict access on the HMD, and therefore, educating participants on what was available prevented adverse outcomes with use of a module they had not been educated on. This was also due to the intent to collect subjective information from participants on what they found beneficial for their pain, whether FDA-registered or not, as well as ensure focus on real-world use as opposed to a regimented protocol. During week 2 of the study (week 1 of using the VRT), participants were encouraged to use VRT once a day for no more than 15 min to assess for adverse reactions. If tolerated after that first week, they were permitted to use at intervals and frequencies of their choosing, with instructions not to exceed 20 min per session to ensure tolerability. At the conclusion of the study, participants completed a qualitative survey to obtain the participants’ perceived benefits, which may not always correlate with reduction in pain sensitivity (see Supplementary Table S4).

The protocol for this study was reviewed and approved by the University of Nevada, Reno Institutional Review Board (IRB) (1704801-1). The authors declare that the research was conducted in the absence of any commercial or financial relationships that could be construed as a potential conflict of interest. Funding was provided by Whole Health at VA Sierra Nevada Health Care System to support rental of the headsets for the study period. The views and opinions expressed in this article are those of the authors and do not reflect the views of any agency of the US government, including the Department of Veterans Affairs.

## Results

Objectively, minimal benefit was noted in the improvement in overall pain, improving less than 13% from baseline to the end of the study when looking at average pain scores. However, there are more impressive improvements noted when specifically analyzing the pain scores preimmersion and postimmersion, which showed an average of 22% reduction in pain intensity on the DVPRS scale (see Supplementary Table S3), emphasizing that users found benefit while actively using the device, but no evidence of significant benefit over time. This information was collected in the pain diary and analyzed after study completion; in future analyses it would be beneficial to assess duration of this benefit, however, this was not done in this study. Due to the small number of patients and lack of control group, these authors are not able to generate the statistical significance of these findings.

In addition to modest improvements in pain intensity, participant's reported profound subjective improvements. Included below are direct participant quotes, as these authors feel that the impact is best heard in the participants own words. In the area of mindfulness, participants made statements such as “nothing bothers me; I’m more open-minded,” “[my provider] thought I fell asleep but I was just relaxed and using my deep breathing,” and “my mood is improved, less worried and more relaxed.” Participants also reported benefits in other areas of their health; one participant noted “it has not helped with a decrease in pain but has helped with endurance and focusing on the breathing; it is like using a spirometer.” Another reported “I’m getting some relief and sleeping a little better.” Yet another reported an improvement in mood and stated that “my wife and children think I’m a nicer person [after using VRT].” The experience of immersion was described as “enjoyable” and “profoundly satisfying.” One participant identified valuable limitations of VRT, stating “VR helps to work on focus, but the pain does not go away. It can help with specific parts of the body but not everywhere.” From the results of the Likert survey (see Supplementary Table S4), the participants felt that the use of VR reduced their pain, decreased stress, and improved their mood. They also reported it useful and enjoyable to be able to use at home, and would overall recommend the use of VRT to other Veterans.

The authors would also like to note that there were no injuries reported, two participants reported mild motion sickness, which was self-limiting and resolved when the headset was removed, and all headsets and pain diaries were returned and scanned into the medical record. All headsets were returned in working order.

Four participants decided not to continue to participate and withdrew. All 4 of these participants had unique reasons for withdrawing: one’s health was deteriorating; one Veteran was finding little benefit and wanted to focus on the present chronic pain regimen; a third Veteran had home-based internet but was not able to adequately set up the technology; and one had an accident requiring hand surgery, which made the use of the VR system too complicated. It should be noted, however, that all 4 of these Veterans expressed positive benefit and experience when given an in-clinic VR demonstration before signing the consent. All were excited to participate and explore the use of VR to help manage their chronic pain.

## Discussion

The benefits of VRT have been previously demonstrated for both acute and chronic pain,^12^ however, at the time this study protocol was created, the majority of available evidence on the use of VRT took place in highly monitored settings, specifically inpatient settings,^13–14^ which is not reflective of the setting in which most pain care is provided. Largely, pain care is done in the outpatient setting, with patients presenting for assessment at routine intervals, but daily management is done by the individual, such as taking prescribed medications on a routine basis or participating in self-directed exercise. Given the established benefits of VRT on pain intensity but lack of use in a real-world setting, these authors set out to assess if VRT could be safe and efficacious for use in the outpatient setting to reduce pain intensity. Similar to the authors of the recent Letter to the Editor,^15^ these findings are not large enough to draw statistical significance or dictate clinical recommendations, but rather serve as a first step in addressing safety considerations for the use of VRT for pain management in a home-based setting.

This study suggests some potential beneficial implications for the use of VRT to be used as an adjunct to traditional pharmacological treatment for pain within the Veteran population. Given that Veterans are disproportionately affected with chronic pain, these authors intended to assess benefits to Veterans with this emerging technology. There is a known disparity in gender of Veterans (females only making up 10% of the overall Veteran population),^16^ and so, these authors intentionally ensured recruitment of female Veterans for this analysis to ensure representation.

Recognizing that this analysis was not large enough to draw statistical significance on reductions in pain intensity with the use of VRT, it does suggest relative safety. In addition, many participants also reported subjective benefits on their well-being, sleep, and mood. This suggests that some patients may see a tremendous positive benefit from the use of a multimodal treatment plan, including VRT, to treat their pain. In addition, the use of VRT in the outpatient setting allows Veterans to have on-demand access to patient-centered approaches for managing their pain when and where they desire with a low risk of side effects. The present findings are not large enough to draw statistical significance or dictate clinical recommendations, but rather serve as a first step in addressing safety considerations for the use of VRT for pain management in a home-based setting.

## Limitations

While the pain diary aimed to collect as many pain-related endpoints as possible, the use of the pain diary varied among participants; some participants logged multiple times per day, and some only a handful of times throughout the study. While this may limit the analysis of some data, the authors feel this reflects the real-world nature of the use of VRT. Despite inconsistent use, participants still overall reported improvement in their pain.

Given the low number of participants, these authors are unable to find statistical significance in the data obtained by this study. Initial intent of this analysis was to assess improvements in pain intensity utilizing patient-reported pain on an 11-point scale in an attempt to objectify pain improvement as is often done in research for treatments for pain, with significance set at 30% to reflect that of other pain-related research. While the intervention demonstrated benefit in pain score immediately after immersion, improvement in the average reported pain score overall was negligible. This highlights the gaps in this method of analysis, as participants overwhelmingly reported that they found improvements in their pain in the Likert survey, despite this not being reflected in their reported pain scores. This is similarly found for mood, which was reported as improved in the Likert study despite analysis from the diary entries showing an overall reduction in mood score (worsened mood). While the tools used to assess pain intensity and mood were validated tools, the subjective findings related to quality of life, well-being, pain interference, and sleep were by patient report and did not use validated tools to assess, and therefore may be suggestive of benefit but cannot be assessed for significance in this study. Future studies should include more robust assessment and analyses of these components, to better understand a patient’s perception of the benefits of the intervention. There is more to the pain experience than just pain intensity, and these other factors, such as pain interference, catastrophizing, well-being, sleep, and mood, should continue to be explored to better understand the totality of the benefits from VRT.

An important consideration to highlight is that, due to a large focus on real-world utility and benefit, particularly important in such an unprecedented time, the structure of this analysis would be difficult to replicate. Patient education and encouragement of outcomes are able to be replicated, but it is quite possible that results may vary in future analyses. Given the lack of evidence at the time of safe, beneficial use of VRT outside of a clinic setting for the treatment of pain, the methods of these authors was focused largely on ensuring safety and encouraging real-world use. These authors recognized that with requiring stricter adherence to documentation in pain diary or more regimented selection of modules in the HMD, outcomes could change significantly. Should future analyses desire more specific outcomes related to the device itself as opposed to safety with use, these authors would recommend more strict protocols.

One additional limitation to note is that classification of prescribed medication classes (i.e., was patient on opioid therapy at the time of the analysis) was not collected information, as it was not one of the assessed endpoints; rather it was largely focused on desirability, feasibility, and utility when used outside of the clinic setting. Some participants noted that they were able to utilize less of their breakthrough pain medication, but this was captured as a subjective report only and was not further validated to classify the magnitude of the reduction nor the medication(s) in question. Assessing if any relationship exists between utilization of VRT and medication use for chronic pain would be valuable for future analyses.

This was a very small convenience sample and so no generalizations can be implied in the reported findings. However, given the low degree of side effect and the high degree of positive response from those participants who completed the study, there does appear to be the need to perform larger studies to explore if VRT is efficacious to decrease pain. In addition, there should be additional investigation about what might be the best tool to measure benefit from VRT; some respondents reported a shift in their mood, a decrease in anxiety, and improved sleep, which may be similarly or more impactful on their overall well-being compared with pain. It should be noted that while the intent of this study was for patients to be able to direct their intervention (could choose duration, frequency, and module), this confounds the assessment of benefits given the diversity of the experiences of the individuals. To improve this, future analyses could provide more structured instructions to individuals, if the goal is to assess the benefits of specific modules versus the act of immersion itself. Given these findings and the position that interventional use of VRT fall in line with the use of CIH practices, it could be used as an adjunct for supporting Veteran health and wellness across the continuum.

## Supplementary Material

Supplementary Table S4

Supplementary Article S1

Supplementary Article S2
