# Supplementary material for: Use of Virtual Reality Therapy (VRT) for Home-Based Pain Management, an Observational Cohort Study
Source: J Med Ext Real. 2024 May 2;1(1):93–9. doi: 10.1089/jmxr.2023.0006 (PMC11290039; doi:10.1089/jmxr.2023.0006)
Supplement: Supplementary Table S1 [file jmxr.2023.0006_telepain_supplementary_material_3.21.24.docx]

Supplementary Material

# Supplementary Material

Table 1: Baseline Characteristics

|  | Enrolled | Completed |
| --- | --- | --- |
| Gender | 7 male, 3 female | 3 male, 3 female |
| Age Range | 35-74 years (average age 54) | 35-67 (Average age 51) |
| Race | 3 Hispanic  2 African American  5 Caucasian | 3 Hispanic  1 African American  2 Caucasian |

Table 2: Inclusion and Exclusion Criteria

| Inclusion Criteria | Exclusion Criteria |
| --- | --- |
| - Age 18 years or older - Veteran eligible for health care benefits through Veterans Affairs and enrolled at the VA Sierra Nevada Health Care System - Chronic pain identified by a provider - Reliable access to wireless internet at home | - medical history significant for any of the following:   - motion sickness   - vertigo   - seizures   - recent head/neck surgery   - severe nausea   - impairment of upper extremities   - severe visual disturbance or impairment   - known issues with balance, dizziness, or disturbances with proprioception - unreliable internet access/insufficient bandwidth speeds - intolerability of demonstration with device |

**Table 3: Summary of Findings**

|  | Participant 1 | Participant 2 | Participant 3 | Participant 8 | Participant 9 | Participant 10 | Average |
| --- | --- | --- | --- | --- | --- | --- | --- |
| Average improvement in pain from baseline (%) | 0.32 | 8.33 | -0.51 | 15.99 | 44.78^#^ | 7.75 | 12.77 |
| Improvement in mood from baseline (%) | -5.56 | -4.76 | -12.9 | 3.33 | 27.19 | -11 | -0.62 |
| Average pain reduction pre/post immersion (all weeks) (%) | 27.05 | 65.83 | 0 | 11.67 | 28.13^#^ | 23.53 | 22.03* |
| Number of pre-/post-immersion entries in pain diary (through week 7) | 21 | 58 | 41 | 26 | 52^#^ | 24 | 37 |

^#^Participant 9 utilized the headset for 2 weeks longer than other participants due to scheduling for return of the headset. When averaging only data through week 7 (to mirror that of the other participants) their average reduction pre/post immersion reduction in pain was 28.13%. When including their additional data entries in weeks 8 and 9 that they had the headset, the average was 26.70%. Participant 9 had 52 pain diary entries through week 7, and 72 if including weeks 8 and 9. For average pain improvement, all of Participant 9’s entries were included to capture the scope of the benefit, however this made for slight variability in data analysis.

*Due to the variation in participant utilization of the pain diary, the average reduction in pain intensity pre- and post-immersion is not equal to the average of their individual reductions. The average pain reduction pre/post immersion (all weeks) was calculated weighing each entry individually, as opposed to varying weights based on participant utility of the diary. For participant 8 who utilized the headset for longer than other participants, only their diary entries through week 7 were included in the average.

**Table 4: Likert Scale Results**

| Participant number | VR1 | VR2 | VR3 | VR8 | VR9 | VR10 | Average Score |
| --- | --- | --- | --- | --- | --- | --- | --- |
| Did the use of VR decrease your pain? | 4 | 5 | 3 | 4 | 5 | 5 | 4.33 |
| Did the use of VR decrease my stress? | 5 | 5 | 4 | 4 | 5 | 5 | 4.67 |
| Did I find it useful to use VR at home? | 4 | 5 | 4 | 5 | 5 | 5 | 4.67 |
| Did I find it enjoyable to use VR at home? | 5 | 5 | 4 | 5 | 5 | 5 | 4.83 |
| Did the use of VR improve my mood? | 5 | 5 | 4 | 5 | 5 | 4 | 4.67 |
| Would I recommend VR to other Veterans? | 4 | 5 | 4 | 4 | 5 | 5 | 4.50 |

Likert Scale Scoring: 1 = strongly disagree through 5 = strongly agree

**Article 1: Example Consent**

**WHAT IS THE STUDY ABOUT AND WHY ARE WE DOING IT?**

This study is about the use of virtual reality (VR) goggles to reduce pain for participants living at home in an outpatient setting. It is being funded by the Whole Health Department and is facilitated by the Physical Medicine and Rehabilitation Department (PM & R) at the VA Sierra Nevada Health Care System (VASNHCS). By participating in this study, we hope to learn if this is an effective nonpharmacological approach that helps patient’s to decrease pain. No changes to a participant’s current treatment plan or use of medications are required to participate. The use of the VR goggles will be in addition to the care participants are already receiving.

The purpose of this research is to gather information on the safety and effectiveness of using VR goggles with software that is FDA approved to be used for patients with pain. The goggles and software will be used as designed to manage pain.

**what does the study involve AND HOW LONG WILL IT LAST?**

This research will last about 7 weeks including 1 week to collect baseline data on pain and 6 weeks with the VR goggles to test the effectiveness to impact pain levels. You will be asked to use the VR goggles for 15 minutes for the first week and up to 3 times a day for 15-minute sessions. In addition, you will be asked to collect pain levels before and after the VR immersion and 4 times throughout the day and document this in a pain log.

**what are key reasons you might choose to volunteer for this study?**

A key reason for participating in this study is to assist VASNHCS to evaluate whether VR is an effective nonpharmacological intervention to help decrease pain for patients at home or in an “outpatient” setting. An additional potential benefit is for the participants to experience a decrease in their pain from the use of the VR goggles.

**what are key reasons you might choose not to volunteer for this study?**

VR is a non- invasive intervention and is deemed “minimal risk”.

The key reason you may not want to participate is if you do not have sufficient time to dedicate to using the VR headset as required or completing the needed documentation on a daily basis. This may range from 20 minutes per day in the first week to up to 60 minutes per day in subsequent weeks.

**DO YOU HAVE TO TAKE PART IN THE STUDY?**

If you decide to take part in the study, it should be because you really want to volunteer. You will not lose any services, benefits, or rights you would normally have if you choose not to volunteer.

**what if you have questions, suggestions or concerns?**

The person in charge of the study is XXXXXXXX Principal Investigator for VASNHCS. If you have questions, suggestions, or concerns regarding this study or you want to withdraw from the study her contact information is XXX-XXX-XXXX or XXXXXXX.email.com.

**RESEARCH DETAILS**

**WHAT IS THE PURPOSE OF THIS STUDY?**

With this research, we hope to learn if it is effective and practical to have participants utilize VR in their home to help manage their pain. VR is an evidence-based practice that has been shown to decrease pain in the inpatient environment within multiple facilities. VASNHCS has demonstrated that VR works in the inpatient setting to decrease pain for VASNHCS Veterans. It is hypothesized that Veterans living at home may also benefit from the use of this technology to help decrease their pain.

**HOW LONG WILL I BE IN THE STUDY?**

This research study is expected to take approximately 10 weeks. Your individual participation in the project will take 7 weeks.

**WHAT WILL HAPPEN AND WHAT CAN I EXPECT IF I TAKE PART IN THIS STUDY?**

You will receive a VR headset for 6 weeks to determine if use of this intervention leads to a subjective decrease in pain. You will use the headset in your home. The Defense and Veterans Pain Rating Scale (DVPRS) detailing pain levels from 0 to 10 will be used to measure pain, which mirrors the pain scale used in the outpatient pain clinic. During the intake process, you will receive a copy of the DVPRS scale and instructions for its use.

Prior to receiving the headset, you will be asked to document your pain 4 times daily for one week (upon waking, at lunch, dinner, and at bedtime) to determine a baseline for your pain levels. After receiving the headset, you will be directed to use VR at least once daily for a dose of 15 minutes. You will be required to keep a daily pain journal to document your outcomes.

There are two FDA approved modules that reside on the VR headset that will be used for this project. The modules are approved for over-the-counter use. The two applications are called Luna, which is based on the principals of cognitive behavioral therapy, and Relax8, a meditation-based application. For each application, you will enter your pain score prior to and immediately following the immersion. In addition, the headset will track the duration and frequency of use and transmit that data to the VR Clinician. The data transmitted does not contain any protected health information (PHI) and will only be accessible to the VR clinician.

In the second and third week of the study, if you report no side effects from the use of the headset, you may add a second and third daily dose from either Luna or Relax8 or from the media center which has a selection of immersions including travel and nature scenes.

The VR clinician will call you once a week to validate your pain log with the data collected in the headset. At the end of the 6 weeks, the VR Clinician will conduct a final follow up phone call and will ask you questions about your participation in the project. You will then be asked to return the headset and pain logs.

The data collected from you includes: a pain score both pre-and post-immersion for recommended dose(s); any additional doses of VR with the pre-and post-pain score; the type of immersion chosen for recommended and breakthrough doses; daily pain log (4 times daily); any changes to prescribed use of opioids/analgesics; a daily evaluation of mood; any utilization of health care resources during the 6-week study (i.e. hospitalizations); and any missed work days during the study period. At the conclusion of the study, the team will assess if there was a change in opioid usage based on chart review, data collection and evaluation.

**Research team**

XXXXXXXXX XXXXXXX, Principal Investigator

- Leads project
- Administrative oversight
- Participant identification and recruitment

XXXXXXXXX XXXXXXX, Co-principal Investigator and VR Clinician

- Lead clinician, evaluates whether participant meets inclusion criteria
- Provides education/weekly contact
- Assists with technology questions
- Collects and aggregates data
- Acts as main contact for participants

XXXXXXXXX XXXXXXX, Chief of PM&R

- Department sponsor
- Provides medical oversight
- Participant identification and recruitment

XXXXXXXXX XXXXXXX, PhD

- Participant identification and recruitment
- Psychological oversight
- Data evaluation

**Participants Expectations and Responsibilities**

- Use VR headset as instructed.
- Keep your weekly appointments. If you miss an appointment, please contact the VR Clinician to reschedule as soon as you know you will miss the appointment.
- Keep the VR headset in a safe place for your use only, away from children or pets.
- Fill out your pain log as instructed.
- Ask questions as you think of them.
- Return the headset at the conclusion of the study.

**WHAT POSSIBLE RISKS OR DISCOMFORTS MIGHT I HAVE IF I TAKE PART IN THIS STUDY?**

Any intervention has possible risks and discomforts. VR has been used in the inpatient setting at VASNHCS and is deemed “minimal risk”. The main risks or side effects are listed; rare, unknown, or unexpected risks also may occur but are not anticipated.

FALLS

- Use of VR is recommended to be performed ONLY while seated or reclined, VR may cause an altered level of spatial awareness following the conclusion of the session. Falls may occur if you are standing, leaning, or bending while using the headset. The risk of falling is mitigated by using the VR headset for 15 minutes per immersion and ONLY using while seated.

MOTION SICKNESS

- The applications used for the study are intended to be relaxing, they do not simulate rapid movement or experiences that are known to induce motion sickness; however, there is a very low risk for motion sickness to occur. If this does occur, you would be advised to remove the headset and sit quietly until the motion sickness passes. This would be self-limiting and would resolve quickly.

It is not anticipated that there would be any psychological, social, legal, or financial risks that might result from participating in the study.

**Safety Monitoring**

This project has a low appreciable safety risk due to the non-invasive nature of the intervention as well as lack of adverse events seen with previous analyses. At the time of VR education, you will be asked to report any unexpected or unpleasant experience with the headset. In addition, at each weekly phone call, the VR Clinician will ask about any unpleasant sensations as a part of the routine data collection.

There is always a chance that any procedure can harm you. The procedures in this study are no different. In addition to the risks described above, you may experience a previously unknown risk or side effect.

Risks of the usual care you receive are not risks of this study. Those risks are not included in this consent form. You should talk with your health care providers if you have any questions about the risks of usual care.

In addition to the risks outlined above, other common procedures included in the study that may cause discomfort are:

- (1) Questionnaires

Some people become uncomfortable when asked questions about pain and the use of medication to treat it; if, for any reason, you do not wish to answer specific questions or you wish to terminate the session, you will be able to do so.

- (2) Photographs, audiotaping, or videotaping

There will be no photographs, audio tapes, or video tapes made of you as a part of this study.

**WHAT ARE THE POSSIBLE BENEFITS OF THIS STUDY?**

We do not know if you will get any benefits from taking part in this research study. However, possible benefits may include a decrease in your pain levels during immersion with the headset. An additional benefit in participating is to generate data on the use of VR which may help others suffering with pain.

**HOW WILL MY PRIVATE INFORMATION BE PROTECTED?**

Your privacy will be protected during the study.

All private information and identifiers will be removed from the data that is collected. After that removal, the information could be used for future research studies or distributed to another investigator for future research studies without additional informed consent from you or your legally authorized representative. However, this data will not contain PHI and would not be traceable back to you.

Participation will not affect your VA healthcare including your doctor's ability to see your records as part of your normal care and will not affect your right to have access to the research records after the study is completed.

**Health Information Portability and Accountability Act (HIPAA)**

There are rules to protect your private health information. Federal and state laws and the federal medical law, known as the HIPAA Privacy Rule, also protect your privacy. By signing this form, you provide your permission called your ‘authorization,’ for the use and disclosure of information protected by the HIPAA Privacy Rule.

The research team working on the study will collect information about you. This includes things learned from the interventions described in this consent form. They may also collect other information including your name, address, date of birth, social security number, and information from your medical records such as your diagnosis and medication list.

The research team may also need to disclose your health information and the information it collects to others as part of the study progress. Others may include Institutional Review Board, Food and Drug Administration, Office (FDA), University of Nevada, Reno Institutional Review Board, the VA Office of Research Oversight (ORO), and the Government Accountability (GAO).

You can revoke this authorization, in writing, at any time. To revoke your authorization, you must write to the Release of Information Office at this facility or you can ask a member of the research team to give you a form to revoke the authorization. Your request will be valid when the Release of Information Office receives it. If you revoke this authorization, you will not be able to continue to participate in the study. This will not affect your rights as a VHA patient to treatment or benefit outside of the study.

If you revoke this authorization, Ms. XXXXXX and her research team can continue to use information about you that was collected before receipt of the revocation. The research team will not collect information about you after you revoke the authorization.

Treatment, payment or enrollment/eligibility for benefits cannot be conditioned on you signing this authorization. This authorization will expire at the end of the research study unless revoked prior to that time.

**WHAT ARE THE COST TO ME IF I TAKE PART IN THIS STUDY?**

You will not be charged for any treatments or procedures that are part of this study. If you usually pay co-payments for VA care and medications, you will still pay these co-payments for VA care and medications that are not part of this study. There is no payment offered for participation in the study.

Note: VA policy prohibits paying human subjects to participate in research when the research is integrated with a patient’s medical care and when it makes no special demands on the patient beyond those of usual medical care. Any payment offered should be commensurate with the time and inconvenience of the participant incurred by the participant that they otherwise would not have incurred, as well as to cover travel expenses.

**WHAT WILL HAPPEN IF I AM INJURED BECAUSE OF MY BEING IN THE STUDY?**

The use of VR has minimal risk associated with its use; however, if in the very rare chance an injury is sustained the VA will provide treatment for research related injury in accordance with applicable federal regulations (38 CFR 17.85). If emergency and ongoing medical treatment is needed due to research-related injury, it will be will be provided as needed.

If you should have a medical concern or get hurt or sick as a result of taking part in this study, call:

**DURING THE DAY:**

XXXXXXXX at XXX-XXX-XXXX and

**AFTER HOURS:**

XXXXXXXX at XXX-XXX-XXXX.

**DO I HAVE TO TAKE PART IN THE STUDY?**

Participation in the study is voluntary. Refusal to take part in the study will involve no penalty or loss of benefits to which the participant is otherwise entitled.

In addition, you may discontinue taking part at any time without any penalty or loss of benefits. You may withdraw at any time and still receive the same standard of care that you would otherwise have received.

**WHO DO I CONTACT ABOUT THIS STUDY IF I HAVE QUESTIONS?**

If you have questions about your rights as a study participant, or you want to make sure this is a valid VA study, you may contact the Institutional Review Board (IRB) at XXX-XXX-XXXX. This is the Board that is responsible for overseeing the safety of human participants in this study.

You may call or email XXXXXXXXXXX, Principal Investigator at XXX-XXX-XXXX / XXXXX@email.com or XXXXXXXXXXX Co-principal Investigator and VR Clinician at XXX-XXX-XXXX or XXXXX@email.com; if you have questions, complaints or concerns about the study or if you would like to obtain information or offer input.

**WILL I BE TOLD NEW INFORMATION ABOUT THIS STUDY?**

At the conclusion of the study the research team will provide high level findings as to the effectiveness and feasibility to you, if desired. These results will be provided in aggregate and will not disclose your PHI.

**FUTURE USE OF DATA AND RE-CONTACT**

Aggregated and de-identified data may be used for future research. However, it is not anticipated you will be contacted by the research team following the study.

**AGREEMENT TO PARTICIPATE IN THE RESEARCH STUDY**

Dr./Mr./Ms___________________________ has explained the research study to me. I have been told of the risks or discomforts and possible benefits of the study. I have been told of other choices of treatment available to me. I have been given the chance to ask questions and obtain answers.

By signing this document below, I voluntarily consent to participate in this study and authorize the use and disclosure of my health information for this study. I also confirm that I have read this consent, or it has been read to me. I will receive a copy of this consent after I sign it. A copy of this signed consent will also be put in my medical record.

| **I agree to participate in this research study as has been explained in this document.** | | |
| --- | --- | --- |
| _________________________  Participant’s Name | ____________________________  Participant’s Signature | ___________  Date |

**Article 2: Example Pain Diary**

Veteran Name: ________________________________ Last 4:________________

Date(s): Week of ____________________________________________________________________

| Day/Date |  | Pain Level | Mood | Comments |
| --- | --- | --- | --- | --- |
| Day 1  Date: ___________ | Wake Up |  |  |  |
|  | Lunch |  |  |  |
|  | Dinner |  |  |  |
|  | Bedtime |  |  |  |
|  |  |  |  |  |
| Day 2  Date: ___________ | Wake Up |  |  |  |
|  | Lunch |  |  |  |
|  | Dinner |  |  |  |
|  | Bedtime |  |  |  |
|  |  |  |  |  |
| Day 3  Date: ___________ | Wake Up |  |  |  |
|  | Lunch |  |  |  |
|  | Dinner |  |  |  |
|  | Bedtime |  |  |  |
|  |  |  |  |  |
| Day 4  Date: ___________ | Wake Up |  |  |  |
|  | Lunch |  |  |  |
|  | Dinner |  |  |  |
|  | Bedtime |  |  |  |
|  |  |  |  |  |
| Day 5  Date: ___________ | Wake Up |  |  |  |
|  | Lunch |  |  |  |
|  | Dinner |  |  |  |
|  | Bedtime |  |  |  |
|  |  |  |  |  |
| Day 6  Date: ___________ | Wake Up |  |  |  |
|  | Lunch |  |  |  |
|  | Dinner |  |  |  |
|  | Bedtime |  |  |  |
|  |  |  |  |  |
| Day 7  Date: ___________ | Wake Up |  |  |  |
|  | Lunch |  |  |  |
|  | Dinner |  |  |  |
|  | Bedtime |  |  |  |
|  |  |  |  |  |
